# Supplementary material for: Modulating HSF1 levels impacts expression of the estrogen receptor α and antiestrogen response
Source: Life Sci Alliance. 2021 Feb 16;4(5):e202000811. doi: 10.26508/lsa.202000811 (PMC7893817; doi:10.26508/lsa.202000811)
Supplement: Supplementary file 1 [file LSA-2020-00811_SdataF1_FS1_FS3.pdf]

Method to capture images.

The PVDF membranes were sliced into sections based on the expected protein molecular weight prior to incubation with the primary antibody. The sections were positioned to reconstruct the original membrane for imaging. Bright field and chemiluminescence images were captured using a Chemidoc Gel Imaging System. The dashed lines represent the separation between membrane sections. Substrates and exposure times are indicated above each image. Blue boxes represent bands used to build the figures.

Fig. 1

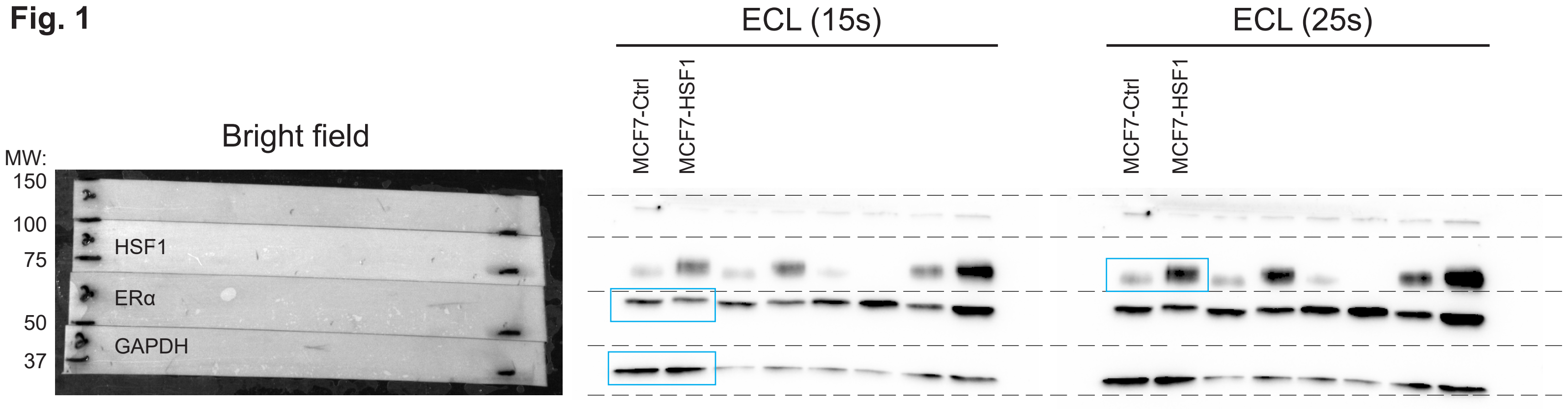

Fig. S1

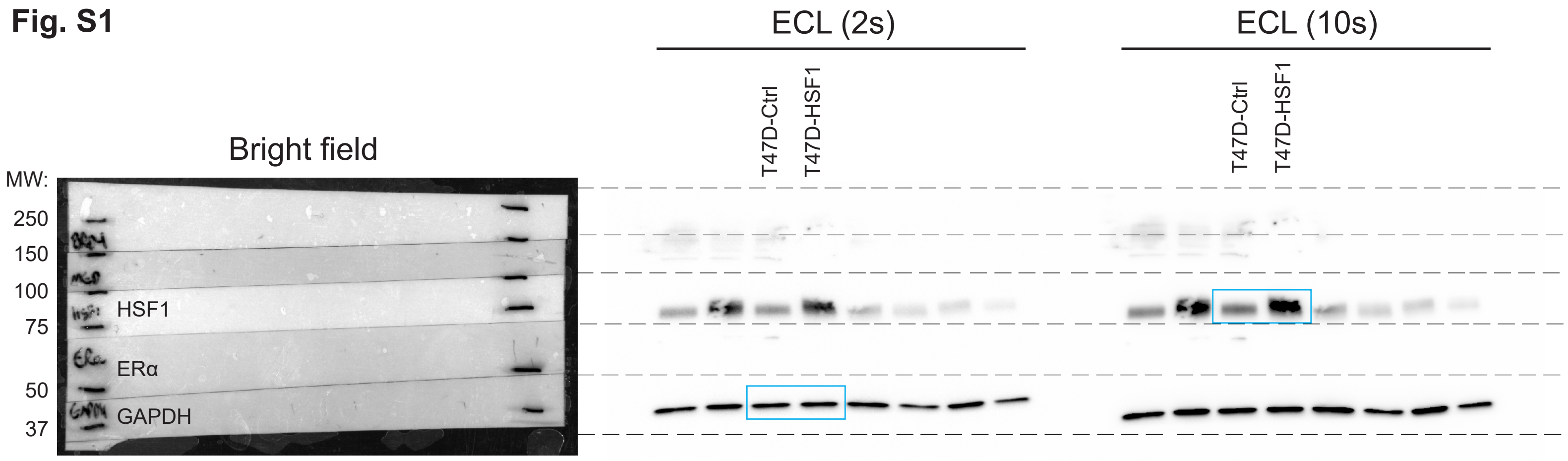

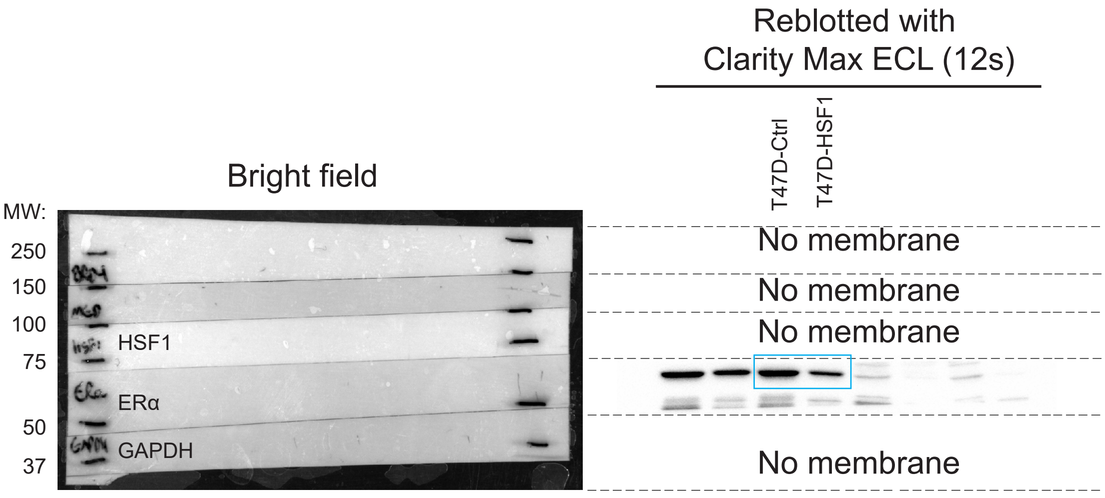

Fig. S3

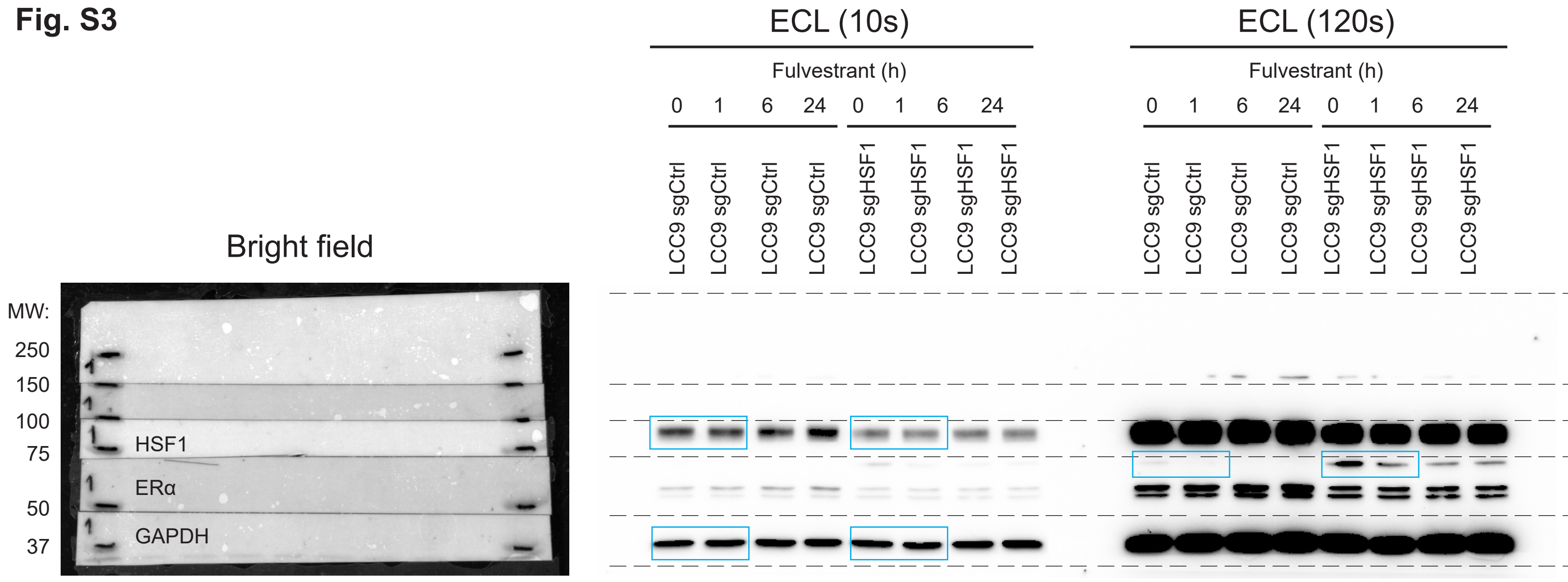

Bright field
MW:
250
150
100
75
50
37
HSF1
ERα
GAPDH
ECL (10s)
ECL (120s)
Fulvestrant (h)
0 1 6 24 0 1 6 24
LCC9 sgCtrl
LCC9 sgHSF1
